# *Arabidopsis thaliana* mitogen-activated protein kinase 6 is involved in seed formation and modulation of primary and lateral root development

**DOI:** 10.1093/jxb/ert368

**Published:** 2013-11-11

**Authors:** J. S. López-Bucio, J. G. Dubrovsky, J. Raya-González, Y. Ugartechea-Chirino, J. López-Bucio, L. A. de Luna-Valdez, M. Ramos-Vega, P. León, A. A. Guevara-García

**Affiliations:** ^1^Instituto de Biotecnología, Universidad Nacional Autónoma de México, Apartado Postal 510-3, 62250 Cuernavaca, Morelos, México; ^2^Instituto de Investigaciones Químico-Biológicas, Universidad Michoacana de San Nicolás de Hidalgo, Edificio A-1′, CP 58030 Morelia, Michoacán, México; ^3^Departamento de Ecología Funcional, Instituto de Ecología, Universidad Nacional Autónoma de México, Ciudad Universitaria, 3er circuito exterior SN, Del. Coyoacán, México D.F. 04510, México

**Keywords:** *Arabidopsis*, embryo development, MAP kinases, MPK6, plant signalling, root development.

## Abstract

Mitogen-activated protein kinase (MAPKs) cascades are signal transduction modules highly conserved in all eukaryotes regulating various aspects of plant biology, including stress responses and developmental programmes. In this study, we characterized the role of MAPK 6 (MPK6) in *Arabidopsis* embryo development and in post-embryonic root system architecture. We found that the *mpk6* mutation caused altered embryo development giving rise to three seed phenotypes that, post-germination, correlated with alterations in root architecture. In the smaller seed class, mutant seedlings failed to develop the primary root, possibly as a result of an earlier defect in the division of the hypophysis cell during embryo development, but they had the capacity to develop adventitious roots to complete their life cycle. In the larger class, the MPK6 loss of function did not cause any evident alteration in seed morphology, but the embryo and the mature seed were bigger than the wild type. Seedlings developed from these bigger seeds were characterized by a primary root longer than that of the wild type, accompanied by significantly increased lateral root initiation and more and longer root hairs. Apparently, the increment in primary root growth resulted from an enhanced cell production and cell elongation. Our data demonstrated that MPK6 plays an important role during embryo development and acts as a repressor of primary and lateral root development.

## Abstract (precis)

*Arabidopsis* MAP kinases are considered to have redundant functions. However, through a detailed phenotypic analysis, we demonstrated that MPK6 loss-of- function cause severe defects in embryo development, which are closed related with alterations in post-germination root development

## Introduction

Mitogen-activated protein kinase (MAPK) cascades are signal transduction modules that are highly conserved in eukaryotes (Zhang *et al.*, 2006). A MAPK module consists of at least three kinases: a MPKKK, a MPKK, and a MPK, which activate downstream targets by phosphorylation. The last kinase of the module, a MPK, is able to phosphorylate several substrates, including transcription factors, to regulate gene expression (Andreasson and Ellis, 2009). MAPKs are known regulators of biotic and abiotic stress responses, hormone perception, and developmental programmes (Colcombet and Hirt, 2008; Suarez-Rodriguez *et al.*, 2010).

The *Arabidopsis* genome encodes 20 different MPKs (MAPK Group, 2002), from which MPK3, MPK4, and MPK6 play important roles both in stress and developmental responses (Colcombet and Hirt, 2008). In particular, MPK6 has been found to participate in bacterial and fungal resistance (Nuhse *et al.*, 2000; Asai *et al.*, 2002; Menke *et al.*, 2004; Wan *et al.*, 2004; Zhou *et al.*, 2004; Zhang *et al.*, 2007), in mutualistic interactions (Vadassery *et al.*, 2009), in priming of stress (Beckers *et al.*, 2009), and in regulation of plant architecture (Bush and Krysan, 2007; Müller *et al.*, 2010).

Functional redundancy is common among MAPKs. Particularly, MPK3 and MPK6 participate in biotic and abiotic stress resistance as well as in developmental processes (Lee and Ellis, 2007; Hord *et al.*, 2008; Lampard *et al.*, 2009; Liu *et al.*, 2010). MPK4/MPK6 and even MPK3/MPK4/MPK6 have been shown to act redundantly in osmotic, touch, wounding, and defence responses (Ichimura *et al.*, 2000; Droillard *et al.*, 2004; Meszaros *et al.*, 2006; Brader *et al.*, 2007). MPKs are proposed to act through common downstream targets and upstream activators (Feilner *et al.*, 2005; Merkouropoulos *et al.*, 2008; Andreasson and Ellis, 2009; Popescu *et al.*, 2009), but the interaction of these pathways is poorly understood. The MPK6 loss-of-function mutant displays alterations in the embryo and early root development, indicating that, at least for these processes, the function of this kinase cannot be substituted by any other MPK (Bush and Krysan, 2007; Müller *et al.*, 2010; Wang *et al.*, 2010).

The first evidence demonstrating that MPK6 (and/or MPK3) is involved in embryo development was reported by Wang *et al.* (2007), who showed that *mpk3* ^–/–^ *mpk6* ^–/–^ double mutants die at the embryo stage and a viable double mutant (*mpk6* ^–/–^ MPK3RNAi) is developmentally arrested at the cotyledon stage. In a different study, Bush and Krysan (2007) reported that several development programmes are influenced by MPK6. In that work, it was observed that *mpk6* null mutant alleles had defects in anther and embryo development, and displayed reduced male fertility. The observed *mpk6* phenotypes display variable penetrance, probably influenced by the growth conditions. Additionally, mutations in the *MPK6* gene have been linked to protrusion of the embryo detected in about 7% of the seeds from an *mpk6* homozygous population (Bush and Krysan, 2007).

Post-embryonic root development is regulated by multiple plant hormones, nutrient availability, and environmental signals (Fukaki and Tasaka, 2009; López-Bucio *et al.*, 2003). The primary root (PR) originates from the embryo and gives rise to many lateral roots (LRs) during vegetative growth, and each of these will produce more LRs. The quantity and placement of these structures among other factors determine the root system architecture (RSA), and this in turn plays a major role in determining whether a plant will survive in a particular environment (Casimiro *et al.*, 2003; Dubrovsky and Forde, 2012). A further adaptation to increase water and nutrient absorption is performed by root hairs (RHs). These are long tubular-shaped epidermal cell extensions covering roots and increase their total absorptive surface (Datta *et al.*, 2011). Auxin (indole-3-acetic acid, IAA) is recognized as the key hormone controlling both RSA and RH development, whereas cytokinins are regulators of PR growth and LR formation (Fukaki and Tasaka, 2009; De Smet *et al.*, 2012).

Current challenges are focused on determining the signalling events for which cell identity regulators are connected with hormone receptors to coordinate stress and development responses. Recently, MPK6 was proposed to be involved in early root development, possibly through altering cell division plane control and modulating the production of second messengers, such as nitric oxide (NO) in response to hydrogen peroxide (Müller *et al.*, 2010; Wang *et al.*, 2010). It was observed that *mpk6-2* and *mpk6-3* mutants produced more and longer LRs than wild-type seedlings after application of a NO donor or H_2_O_2_ (Wang *et al.*, 2010). However, the hormonal responses underlying these root alterations and the role of MPK6 in these processes are still unknown. Thus, independent data support the participation of MPK6 in both shoot and root development, but no relationship has been established between embryo and root phenotypes in *mpk6* mutants, nor the impact of earlier root development alterations in the configuration of post-embryonic root architecture.

In this study, we provided physiological and molecular evidence that seedlings defective in two independent *mpk6* mutant alleles showed three distinct classes of seed phenotype, which correlated with alterations in cell division and elongation processes that affected root architecture. These alterations were independent of MPK3. These data indicate that MPK6 is an essential component of early signalling processes linked to proper embryo development and maintenance of *Arabidopsis* RSA.

## Materials and methods

Additional details are available in Supplementary Methods at *JXB* online.

### Plant material and growth conditions

*Arabidopsis thaliana* Heyhn wild-type and mutant plant lines were in the Columbia-0 (Col-0) ecotype. *MPK6* (At2g43790) T-DNA insertion lines (SALK_073907 and SALK_127507) were obtained from the Salk T-DNA collection (Alonso *et al.*, 2003) and provided by TAIR (http://arabidopsis.org). Both mutant lines were described previously as *mpk6-2* and *mpk6-3* (Liu and Zhang, 2004). The *MPK3* T-DNA insertion line (SALK_151594), was kindly donated by Dr Shuqun Zhang from Missouri University, USA (Wang *et al.*, 2007). The transgenic line *ABI4*::*GUS* (Söderman *et al.*, 2000) was kindly provided by Dr Ruth Finkelstein from the University of California, USA. This marker gene was introduced into the *mpk6-2* background by crossing homozygous plants. Surface-sterilized seeds were incubated at 4 °C for 3 d to break dormancy and then grown on agar (0.8%, w/v, Bacto™ Agar, BD Difco, Sparks, MD, USA) solidified 0.2× MS medium (Caisson, Laboratories, Noth Logan, UT, USA) with 1% (w/v) sucrose. Kinetin and IAA were purchased from Sigma (Sigma-Aldrich, St Louis, MO, USA) and added to the medium at the indicated concentration. Seedlings were grown on vertically oriented Petri dishes maintained in growth chambers at 21 °C under a 16:8h light:darkness photoperiod under 105 µmol m^−2^ s^−1^ light intensity. For seed production, plants were grown in Metro-Mix 200 (Grace Sierra, Milpitas, CA, USA) in a growth room at 23 °C under a 16/8h photoperiod and a light intensity of 230 µmol m^−2^ s^−1^.

### Embryo analysis

Wild-type and *mpk6-2* mutant embryos were processed as described previously (Ugartechea-Chirino *et al.*, 2010). Briefly, ovules were dissected from the silique and punched with a needle in order to favour contact between the embryos and the staining solutions. Embryos were fixed for 1–7 d with 50%, v/v, methanol, 10%, v/v, acetic acid. They were rinsed and incubated at room temperature for 30–45min in 1% periodic acid. After a second rinse, they were incubated for 2h in pseudo-Schiff’s reagent (1.9g of sodium metabisulfite in 97ml of H_2_O and 3ml of 5M HCl with 0.1mg ml^–1^ of propidium iododide). Embryos were rinsed again and transferred to a drop of chloral hydrate (80g of chloral hydrate in 30ml of H_2_O) on a microscope slide. Excess chloral hydrate was removed, and the embryos were mounted in Hoyer’s solution (30g of gum arabic, 200g of chloral hydrate, 20g of glycerol in 50ml of H_2_O). Mounted embryos were cleared in Hoyer’s solution for at least a week before confocal imaging.

### Seed size analysis

Dry seeds were measured individually using ImageJ (http://rsb.info.nih.gov/ij) with a set scale tool to establish a 1mm reference on a micrometer image taken with a Nikon SMZ1500 stereomicroscope equipped with a digital SIGHT DS-Fi1c camera. Seed stereomicroscope images were then analysed with ImageJ using the 1mm reference. Seed weight was obtained by weighting a batch of 100 seeds placed in Eppendorf tubes in an analytical balance.

### Growth analysis

Photographs of representative seedlings were taken with an EOS REBEL XSi digital camera (Canon, Tokyo, Japan). The growth of PRs was registered using a ruler. LR number was determined counting all LRs emerged from the PRs under the Nikon SMZ1500 stereomicroscope. LR density, LR primordium (LRP) density, length of cortical cells, LR initiation index, length of root apical meristem (RAM), length of proliferation domain (PD), length of transition domain (TD), and number of cells in the PD (NC_PD_) were determined on cleared roots as described previously (Dubrovsky *et al.*, 2009; Dubrovsky and Forde, 2012; Ivanov and Dubrovsky, 2013). Position of the most distal (rootward) LRP and the most distal LR as well the number of LRPs in the LR formation and the branching zones was determined on cleared root preparations under a Zeiss Axiovert 200M microscope (Zeiss, Oberkochen, Germany), equipped with differential interferential contrast optics. Cortical cell length was determined for 10 cells per root on cleared preparations using an ocular micrometer. Images of RHs and etiolated seedling images were taken under a Nikon SMZ1500 stereomicroscope equipped with a digital SIGHT DS-Fi1c camera. RH density (number of RHs mm^–1^) and RH length were determined from roots mounted in H_2_O on microscope slides and observed under a Zeiss Axiovert 200M microscope. Cell production rate (CPR) was calculated with the equation CPR=*Vl*_*e*_^*–1*^, where *V* (μm h^–1^) is the rate of root growth during the last 24h before the termination of the experiment and *l*_e_ (μm) is the length of fully elongated cortical cells, whereas cell-cycle duration (*T*, hours) was calculated with the equation *T*=(NC_PD_×*l*_e_ *×*ln2)/*V* ^*–1*^ in accordance with Ivanov and Dubrovsky (1997). This method is applicable to steady-state growing roots. One condition of steady-state growing roots is a linear increase in the root length. We analysed root growth during the last 24h in seedling samples 5 and 8 d after germination (DAG) and found that at both time points the growth in both the mutant and the wild-type was stabilized (see Results). Another condition was a constant number of cells in the meristem (Ivanov and Dubrovsky, 1997). As the transition domain of the RAM has not been defined previously, the number of meristematic cells in the cited work corresponds to the NC_PD_ in the current study. To verify if the NC_PD_ was constant during the analysed time periods, we estimated this parameter in samples at *t*_0_ (24h prior to termination of the experiments) and found no statistical differences in the NC_PD_ within the same genotype at *t*_0_ and at final time points. This preliminary analysis permitted us to apply the above equation for estimation of average cell-cycle time in the PD. Criteria for defining the PD and TD have been described (Ivanov and Dubrovsky, 2013). Briefly, the PD comprises cells that maintain proliferation activity and the TD comprises cells that have very low probability of cell proliferation but grow at the same rate as cells in the PD and have not yet started rapid elongation. As no marker lines have yet been proposed to identify these domains, we determined the domains based on relative changes in the cell lengths analysed on cleared root preparations. In the PD, the cell length commonly varies no more than 2-fold, and in the TD cells are longer than the longest cells in the PD. The distance from the quiescent centre to the point where a cortical cell becomes longer than the longest cell in the PD was considered to be the border between the PD and the TD. In the elongation zone, the cell length starts steadily to increase simultaneously in all tissues. The point where this increase can be observed was defined as the distal (rootward) border between the TD and elongation zone. All measurements were done with an ocular micrometer.

All experimental data were analysed statistically with SigmaPlot 11 (Systat Software, San Jose, CA, USA). Student’s *t*-test or Tukey’s post-hoc test were used for testing differences in growth and root developmental responses, as indicated. The number of independent experiments in each case is indicated in the corresponding figure legend.

## Results

### Mutation of the *MPK6* gene causes three distinct and stable seed phenotypes

**Fig. 1.**
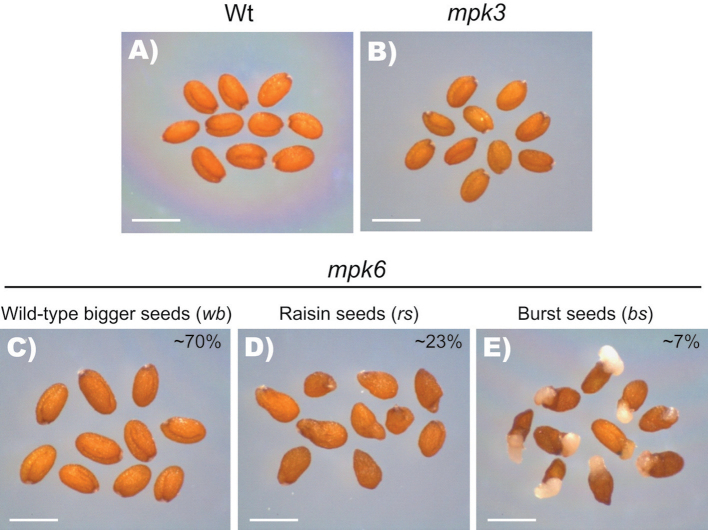
*MPK6* mutation causes three different seed phenotypes. (A, B) Seeds from wild-type plants (Wt, Col-0) (A) and *mpk3* mutant (B) are shown for comparison to stable and distinguishable *mpk6* mutant seed phenotypes. (C) Seeds resembling the wild type but with a bigger seed phenotype (*mpk6wb*). (D) Seeds displaying a rough coat raisin-like seed phenotype (*mpk6rs*). (E) Embryos protruding from the seed coat burst seed phenotype (*mpk6bs*), described previously by Bush and Krysan (2007). For the pictures, seeds from each class were pooled, but the proportion of each phenotype, obtained from 1000 analysed *mpk6* seeds through several generations, is indicated. Bars, 500 µm.

**Fig. 2.**
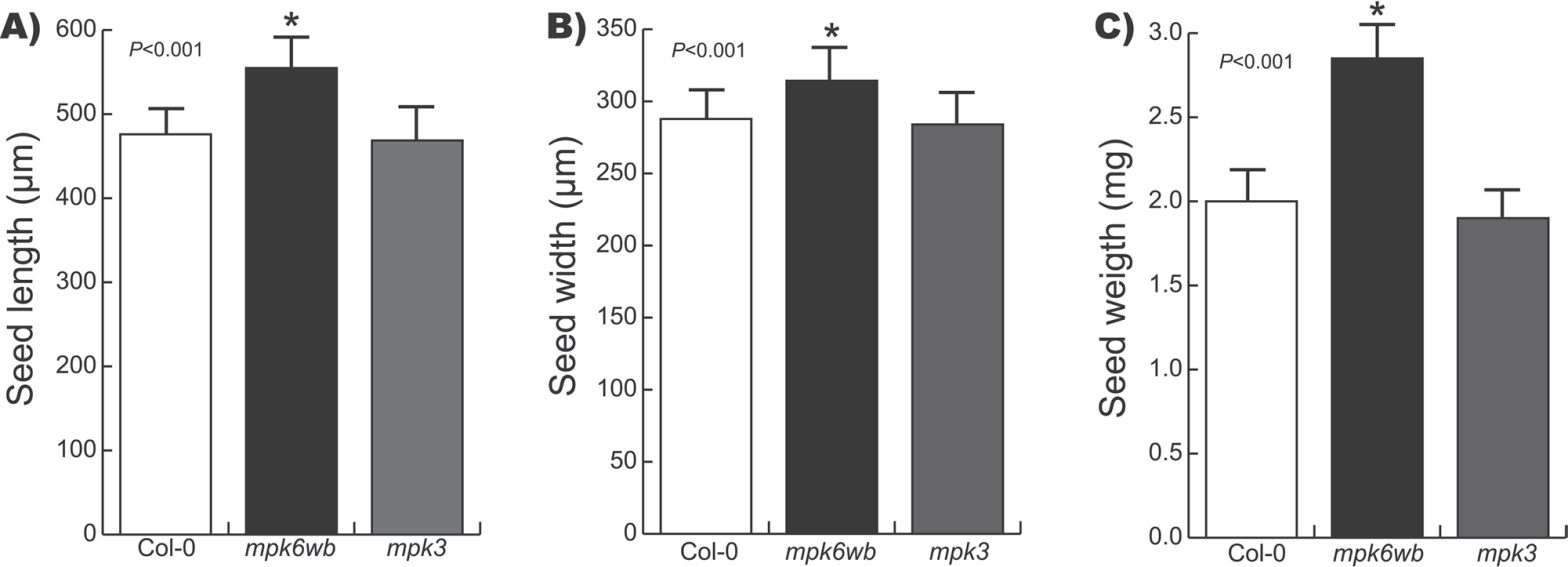
*mpk6* mutant produces seeds bigger than the wild-type. The *mpk6* seeds were longer (A), wider (B), and heavier (C) than wild-type (Col-0) and *mpk3* seeds. Error bars represent the mean ±standard error (SE) from 500 seedlings analysed at each line. Asterisk indicates Student’s *t*-test statistically significant differences at *P* values indicated.

Through a careful phenotypic analysis of two independent *mpk6* T-DNA insertion null mutant lines (SALK_073907 and SALK_127507) (Supplementary Fig. S1A at *JXB* online), we corroborated that the protruding embryo phenotype, previously described by Bush and Krysan (2007), was present in the homozygous seed populations from both mutant alleles. Closer inspection of the seeds from these mutants showed three segregating phenotypically distinctive classes. In the larger class (~70%, *mpk6wb*/*w*ild-type-like *b*igger seeds) the lack of MPK6 did not cause any evident alteration in seed morphology, but the seeds were significantly bigger than those from wild-type plants (Figs 1C and 2). The second class (~23%, *mpk6rs*/*r*aisin-like *s*eed phenotype) included seeds with rough coats (Fig. 1D). Finally, the smaller class (~7%, *mpk6bs*/*b*urst *s*eed phenotype) included seeds with protruding embryos from the seed coat (Fig. 1E). It is important to point out that the rough coat phenotype was not uniform, as we observed some seeds that looked more affected than others (Fig. 1D). However, in this study, all of them were pooled together within the same class. In contrast to the heterogeneous phenotype of the rough coat seeds, the other two seed phenotypes were clearly recognized. To determine whether all three *mpk6* seed phenotypes were linked to the *MPK6* mutation, we performed crosses between a homozygous *mpk6-2* mutant with pollen from wild-type (Col-0) plants. In the F1 progeny of these crosses, no phenotypic alterations were evident. Interestingly, in seedlings from all three different seed classes obtained from *mpk6* homozygous mutant populations, MPK6 activity was absent, and this was observed consistently in at least three subsequent generations of homozygous *mpk6* seedlings from the referred seed phenotypes (Supplementary Fig. S1B).

For a better inspection of seed structure, a *pABI4::GUS* transgene encoding β-glucuronidase (GUS), expressed in embryos (Bossi *et al.*, 2009; Söderman *et al.*, 2000), was introduced into the *mpk6-2* homozygous line. We found that in homozygous *mpk6 pABI4::GUS* F3 populations, all three seed phenotypes were present (Supplementary Fig. S2, at *JXB* online). Previous studies have demonstrated redundancy between MPK6 and MPK3 (Lee and Ellis, 2007; Hord *et al.*, 2008; Lampard *et al.*, 2009; Liu *et al.*, 2010). However, a null *mpk3* mutant allele (SALK_100651) did not display any distinguishable seed phenotype when grown side by side with wild-type or *mpk6* seedlings (Figs 1B and 2), nor was the exacerbated MPK3 activity on *mpk6* seedlings (Supplementary Fig. S1B) able to compensate the *mpk6* phenotypes. Therefore, we concluded that the defects observed in seed morphology were caused specifically by a mutation in *MPK6* and they were apparently independent of *MPK3*.

### *mpk6* seed phenotypes are linked to root developmental alterations

**Fig. 3.**
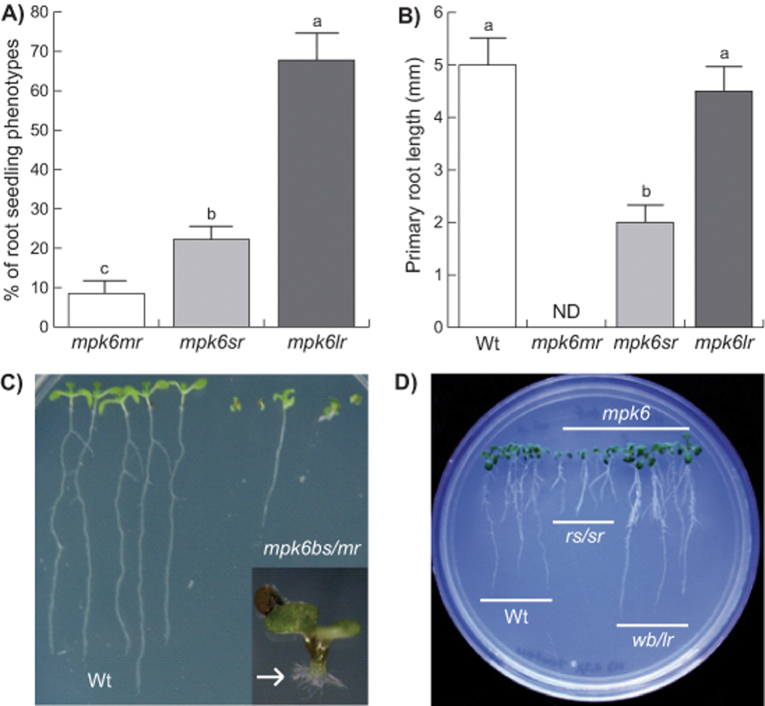
*mpk6* mutant displays three different root phenotypes. The *mpk6* mutant displayed three root phenotypes each related to the seed morphology: seedlings lacking PR (minus root; *mpk6mr*), seedlings with short roots (*mpk6sr*), and seedlings with PR longer than wild-type root (*mpk6lr*). (A) Proportion of 6 DAG seedlings in each *mpk6* root mutant class.(B) PR length in 2 DAG wild-type (Wt) and the three *mpk6* root mutant classes seedlings. Notice that in this developmental stage the root length of the later *mpk6lr* phenotype is similar to that of the wild-type. Error bars represent the mean±SE from data obtained from three independent experiments, each performed with 120 (A) or 100 (B) seedlings. Different letters on the bars indicate Tukey’s post-hoc test statistical difference at *P≤*0.001. (C) Seedlings lacking PR (minus root) developed from *mpk6bs* seeds (*mpk6bs/mr*); some of these seedlings were able to produce adventitious roots (white arrow). (D) Seeds at 6 DAG *mpk6rs* (*rs/sr*) and *mpk6wb* (*wb/lr*) develop shorter and longer PRs compared with the wild-type roots.

To analyse whether the observed alterations in *mpk6* seeds affected post-germination development, we compared the early seedling growth of wild-type and *mpk6* homozygous mutant populations. Initially, we included *mpk3* seeds in our analysis, but we did not find any phenotypic alteration in *mpk3* mutant seeds or root seedlings (data not shown). Inspection of seedlings at 2 DAG demonstrated that it was possible to differentiate three different root phenotypes within the *mpk6* seedlings. Around 70% of the population analysed displayed PRs of greater length than WT seedlings (*l*onger *r*oot; *mpk6lr*). Additionally, roughly 20% of the seedlings displayed *s*hort *r*oots (*mpk6sr*), whereas around 10% of the seedlings did not develop PRs (*m*inus *r*oots; *mpk6mr*) (Fig. 3A, B). Although a previous report has already documented defects in root formation in the *mpk6-2* (SALK_073907) mutant (Müller *et al.*, 2010), no further analysis of these morphological alterations in the root architecture or their relationship with earlier embryonic alterations was performed. Interestingly, the proportion of each of the three root phenotypes correlated with those proportions observed from the seed phenotypes described previously (Figs 1C–E and 3A), suggesting that they may be related.

**Fig. 4.**
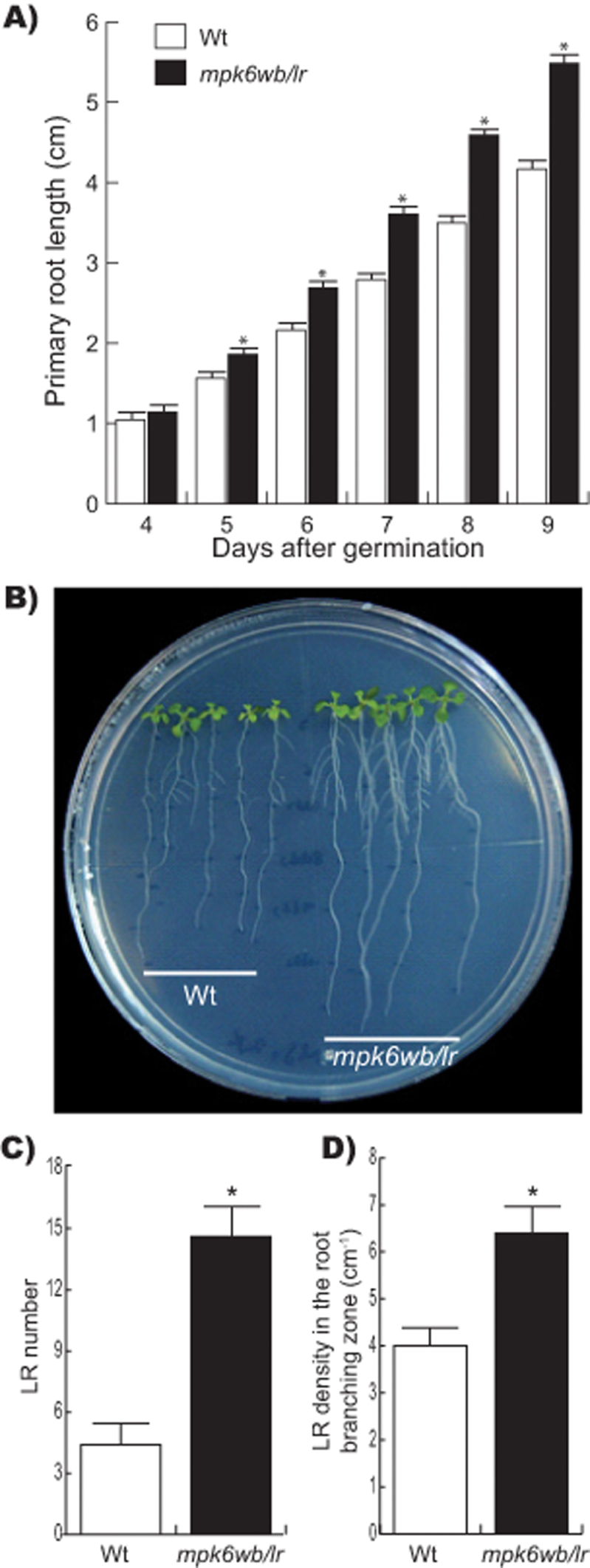
*mpk6* mutant has altered primary root development, more LRs and higher LR density. (A) Primary root length changes with time. Starting from 5 DAG, statistically significant differences were observed between wild-type (Wt) and *mpk6wb/lr* PR length. Error bars represent the mean ±SE from 30 seedlings analysed at each indicated DAG. The experiment was repeated three times with similar results. Asterisk indicates Student’s *t*-test statistically significant differences at *P≤*0.001. (B) Representative photograph of wild-type and *mpk6wb/lr* 8 DAG seedlings. Notice that *mpk6wb/lr* seedlings had longer PRs, and more and longer LRs than the wild-type seedlings. (C, D) LR number (C) and LR density (D) were obtained from the root branching zone of wild-type and *mpk6wb/lr* mutant. Error bars in (C) represent the mean ±SE from 60 seedlings analysed at 8 DAG from three independent experiments and in (D) represents the mean ±SE from 12 seedlings at 6 DAG from two independent experiments. Asterisk indicates Student’s *t*-test statistically significant differences at *P≤*0.001.

To analyse if *mpk6* mutant seed phenotypes had any effect on the post-germination development, the different classes of seed from this mutant were separated and germinated independently. The root morphology from each seed population was then compared with that of wild-type seedlings. Surprisingly, a high proportion of the *mpk6bs* seeds germinated *in vitro*, indicating that, in spite of the protrusion from the seed coat, these embryos were viable (Fig. 3C). However, around 80% of these germinated seedlings failed to develop PRs and most of them died a few days after germination. Those seedlings that survived all developed adventitious roots (Fig. 3C, inset) and were able to complete their life cycle and produce seeds. The progeny from these *mpk6bs* seedlings segregated again into all three seed phenotypes shown in Fig. 1 with similar proportions (data not show). Marked differences were also observed in root development of the seedlings derived from the *mpk6wb* and *mpk6rs* seed types when compared with wild-type seedlings. Seedlings at 6 DAG derived from *mpk6rs* displayed shorter roots than the wild-type, and those derived from *mpk6wb* had longer roots (Fig. 3D). In particular, analysing the rate of growth of the PR of *mpk6wb/lr*, we found that, starting from 5 DAG, it was greater in the mutant than in wild-type seedlings (Fig. 4A). The data described so far clearly demonstrated that MPK6 plays an important role in root development and that these root phenotypes are linked with particular seed phenotypes. Besides a longer root, *mpk6wb/lr* seedlings grown *in vitro* also clearly developed more LRs (Fig. 4B). We next decided to explore the participation of MPK6 in LR development, quantifying the number of LRs and LR density in *mpk6wb/lr* and wild-type plants. These analyses demonstrated that *mpk6wb/lr* seedlings contained a higher number of LRs and a greater LR density in the root branching zone (Fig. 4C, D). These data indicated that MPK6 acts as a negative regulator of LR formation.

### *mpk6* mutant has embryo development defects

**Fig. 5.**
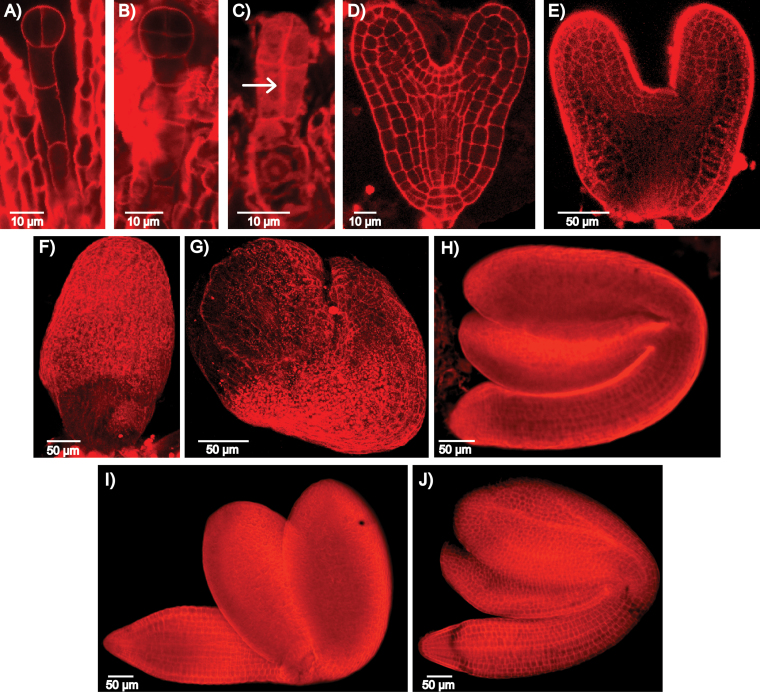
*mpk6* cell organization is affected throughout embryonic development. Cell organization in wild-type (Col-0) and *mpk6* embryos. (A, B) Representative two- to four-cell (A) and eight-cell (B) wild-type embryos during their first three rounds of cell divisions. (C) Two-cell *mpk6* embryo showing ectopic periclinal cell division in the uppermost suspensor cell (arrow). This embryo failed to establish the transversal cell division plane necessary to generate an eight-cell embryo proper. (D) Wild-type heart-stage embryo. (E–G) Immature *mpk6* embryos showing arrested development at the heart (E), torpedo (F), or bent cotyledon (G) stages. (H) *mpk6* embryo with complete embryonic organogenesis at the bent cotyledon stage. (I, J) Representative *mpk6* (I) and wild-type (J) embryos at mature stage. Propidium iodide pseudo-Schiff staining of the cell wall (red) was carried out according to Ugartechea-Chirino *et al.* (2010). Bars are as indicated.

The longer root phenotype of the *mpk6wb/lr* mutant could be a result of differences in germination time compared with that of wild-type. We found that this was not the case, as both wild-type and *mpk6wb* seeds had similar germination times (data not shown) and similar root length during the first days after germination (Figs 3B and 4A). Thus an alternative hypothesis is that the short-root phenotype and the inability to form PRs are associated with defects during embryo development and do not represent alterations in the vegetative root developmental programme. To test this idea, we analysed the morphology of a total of 239 *mpk6* embryos representing all stages of embryonic development from two independent experiments (Fig. 5). During early embryogenesis, the suspensor uppermost cell is recruited to the embryo proper and acquires hypophyseal identity (Jürgens, 2001). Mutant lines defective in generating this cell lineage often produce rootless seedlings (Peris *et al.*, 2010; Jeong *et al.*, 2011). Microscopic analyses of early *mpk6* embryos showed ectopic divisions in the suspensor at the time when the hypophyseal cell should be specified. Seven out of 23 embryos observed between the one-cell and globular stages had ectopic divisions either in the suspensor or in the embryo proper (Fig. 5C). Later during development, 24% of the embryos were arrested at the heart stage embryo and did not proceed to develop hypocotyl and root (Fig. 5E), while 18% showed arrested development but developed hypocotyl and root (Fig. 5F–G) and 71% achieved complete embryonic organogenesis by the bent cotyledon stage (Fig. 5H). Interestingly, at the mature stage, *mpk6* embryos seemed to be bigger than the wild-type (Fig. 5I, J). Considering that *mpk6* developed several short siliques, with few seeds and many abortion events (Supplementary Fig. S3 at *JXB* online), we estimated that the frequency of these *mpk6* embryo developmental patterns correlated roughly with the frequencies observed for the burst, the raisin-like and the bigger phenotypes of mature seeds, respectively.

### MPK6 is involved in the control of RSA

**Fig. 6.**
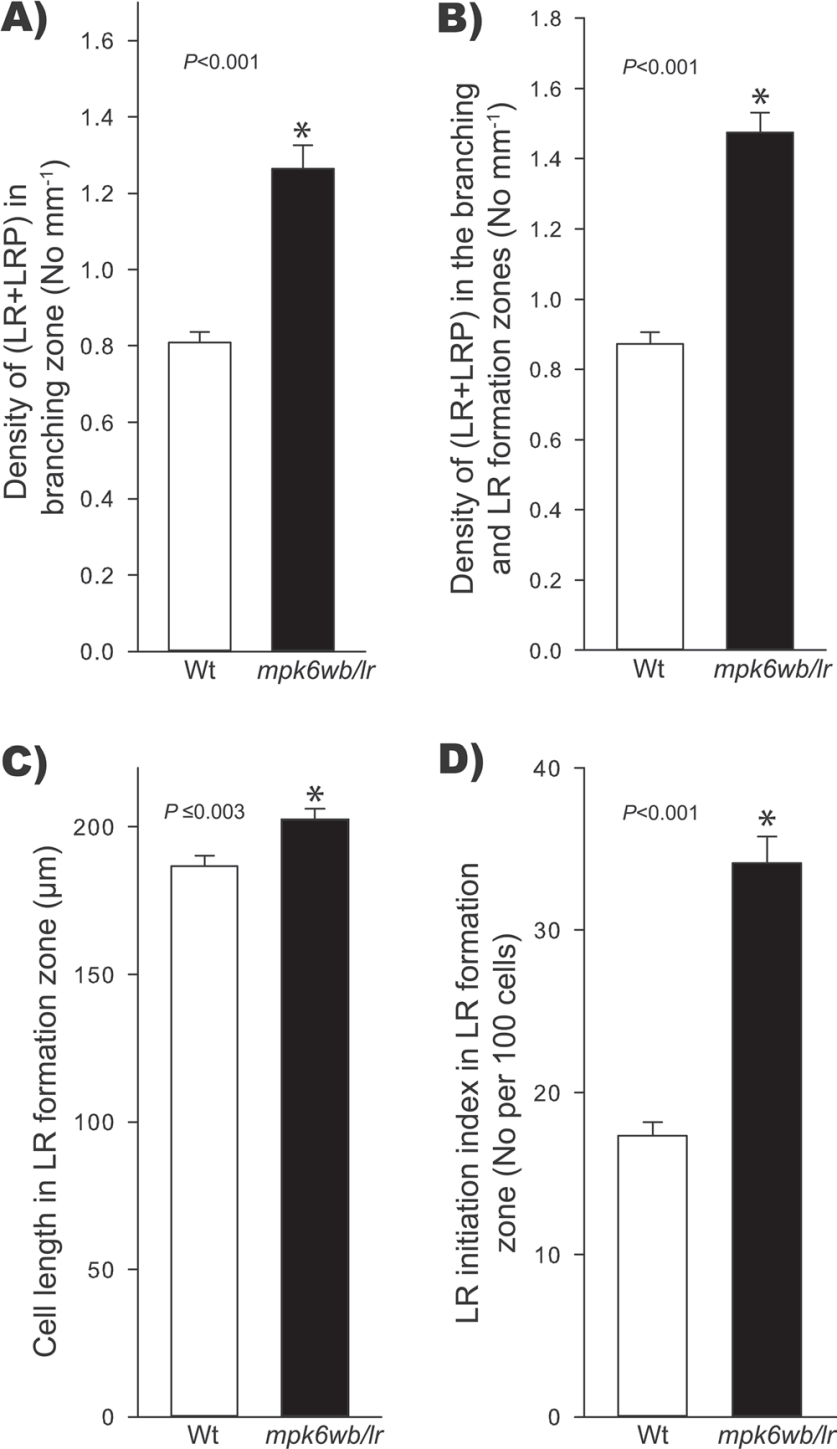
The *mpk6wb/lr* mutant has increased LR initiation. LR formation in wild-type (Wt) and *mpk6wb/lr* seedlings. (A) Density of LRs and LRP in the primary root branching zone. (B) Density of LRs and LRP in branching and in LR formation zones. (C) Cortical cell length in the LR formation zone. For each individual root, 10 fully elongated cortical cells were measured. (D) LR initiation index in the LR formation zone. Error bars represent mean ±SE of 23 roots from two independent experiments. Asterisks mark Student’s *t*-test significant differences at the *P* values indicated.

RSA is an important trait determining plant productivity. At present, little is known about the intrinsic mechanisms that control root growth and branching. To analyse the role that MPK6 has over RSA, we performed experiments to compare PR growth, LR formation, and RH development in wild-type and *mpk6* mutants. As they apparently do not have embryo alterations that can affect the post-germination development, to do this analysis we used only the big seed phenotype that was also associated with long roots (*mpk6wb/lr*). LR number and length were important determinants of RSA and both were affected in the *mpk6wb/lr* mutant (Fig. 4C, D). LRs develop from the parent root through the specification of pericycle founder cells. After activation, these cells undergo repeated rounds of cell division leading to the formation of a LRP that eventually emerges as a new LR (Laskowski *et al.*, 1995; Malamy and Benfey, 1997; Dubrovsky *et al.*, 2001). A strict analysis of LR development must take into account all LR initiation events (Dubrovsky and Forde, 2012). Thus, the densities of all LR initiation events (LR and LRP) in the branching zone and in the branching zone plus LR formation zone (the latter comprises the root portion from the most rootward primordium to the most rootward emerged root) were also analysed. As shown in Fig. 6A, B, both the LR and LRP densities were significantly higher in the *mpk6wb/lr* mutant than in wild-type roots. As the fully elongated cortical cells in the *mpk6wb/lr* mutant could be longer than those from the wild type, the cell length in the LR formation zone (Fig. 6C) and the LR initiation index (Fig. 6D) were also evaluated. The latter parameter permits evaluation of LR initiation on a cellular basis and estimates the number of LR initiation events per root portion comprising 100 cortical cells of average length in a file (Dubrovsky *et al.*, 2009). This analysis also confirmed that LR initiation was significantly higher in the *mpk6wb/lr* mutant compared with wild-type seedlings. These data together strongly supported the conclusion that MPK6 acts as a negative regulator of LR initiation in *Arabidopsis*.

**Fig. 7.**
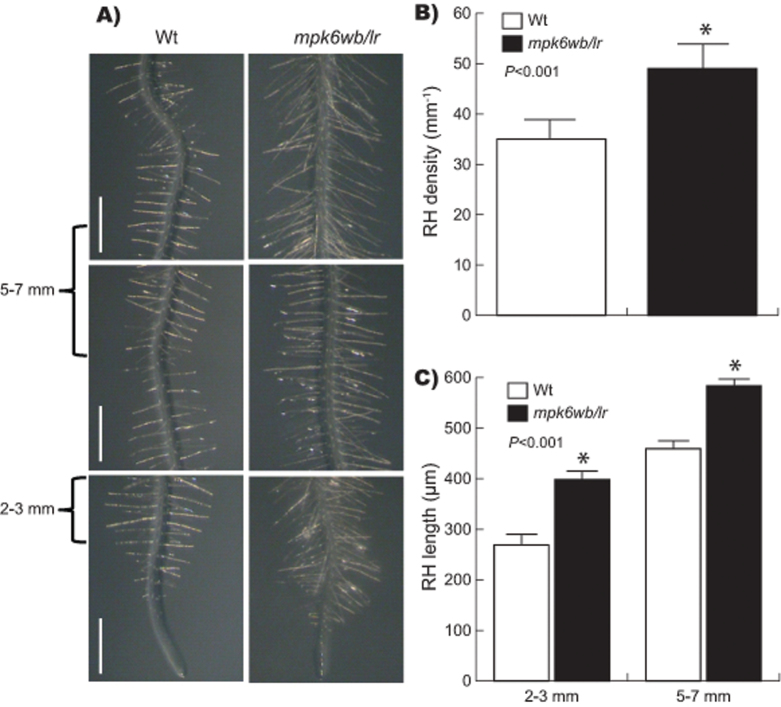
The *mpk6wb/lr* mutant develops more and longer RHs. (A) RHs formed along ~1cm from the tip of the PR from representative 6 DAG seedlings. Bars, 1mm). (B) The RH density in 6 DAG seedlings from 2–3 and 5–7mm root segments measured from the root tip. (C) Comparative quantification of RH length in the same root segments as in (B). Error bars represent the mean ±SE from 30 seedlings in three independent experiments. Asterisks mark Student’s *t*-test significant differences at the *P* values indicated.

RHs differentiate from the root epidermal cells in a cell-position-dependent manner, increasing the total surface of roots (Tominaga-Wada *et al.*, 2011). Based on our previous results, we were interested to determine the effect of the *MPK6* mutation on RH differentiation. As shown in Fig. 7, the total number of RHs was significantly increased in the mutant compared with wild-type seedlings (Fig. 7A, B). These data indicated that the loss of function of MPK6 resulted in more and longer RHs. Moreover, we also found that the length of RHs in two different zones of the PR (2–3 and 5–7mm root portions from the root tip) was also increased in the *mpk6wb/lr* mutant (Fig. 7C). These data also showed an important role of MPK6 in the differentiation and subsequent growth of RHs.

### *mpk6* primary root growth alterations are multifactorial

**Fig. 8.**
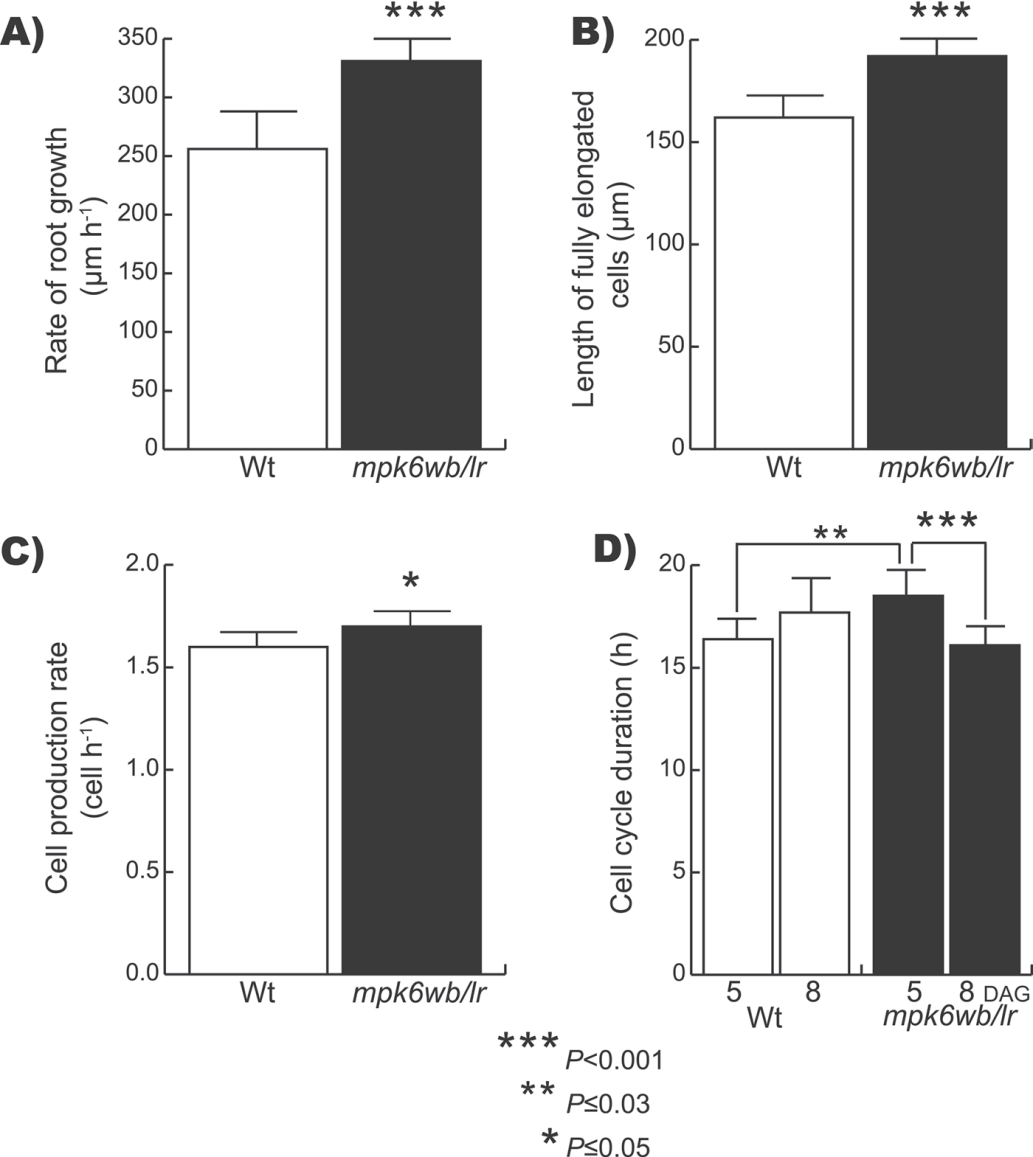
Quantitative analysis of the wild-type (Wt) and *mpk6wb/lr* primary root growth and development. (A–C) Comparison between wild-type and *mpk6wb/lr* of root growth rate (A), length of fully elongated cells (B) and cell production rate (C) from 5 DAG seedlings. Error bars represent the mean ±SE from 24 seedlings in two independent experiments. (D) Cell-cycle duration (*T*) in wild-type and *mpk6wb/lr* at 5 and 8 DAG. In 5 DAG seedlings, *T* is the mean ±SE from 24 roots in two independent experiments. In 8 DAG seedlings, *T* is the mean ±SE from 12 roots in one experiment. Asterisks indicate Student’s *t*-test significant differences at the *P* values indicated.

Cell division, elongation, and differentiation are closely linked cellular processes that determine PR growth. The RAM comprises two different zones: the PD, where high cell proliferation activity and a relatively slow growth takes place, and the TD, where cell proliferation probability is low but cell growth is maintained at a similar level to that found in the PD (Ivanov and Dubrovsky, 2013). After cells leave the RAM, they enter into the elongation zone, where rapid cell elongation takes place. To elucidate the contribution of cell division and elongation to the longer root phenotype of the *mpk6wb/lr* mutant, a detailed morphometric analysis of both PD and TD was conducted on 5 DAG plants. We observed that, while the size of the TD in the *mpk6wb/lr* mutant was 45% greater than that in the wild-type, the PD was 9% greater in the *mpk6wb/lr* mutant (Table 1). We also found that the number of cells, the rate of root growth, the fully elongated cell length, and cell production were also increased in *mpk6wb/lr* PRs compared with those of wild-type (Table 1 and Fig. 8A–C). A significant decrease (13%) in cell-cycle duration over time (5–8 DAG) was found in mutant seedlings (Student’s *t*-test at *P≤*0.001), whereas in the wild type, no changes in cell-cycle duration were found during the same period (Fig. 8D). These results indicated that both cell production and cell elongation have a significant impact on the accelerated root growth found in the *mpk6wb/lr* mutant.

**Table 1.** Wild-type (Col-0) and mpk6wb/lr RAM comparative analysis

Genotype	RAM length (µm)	Difference (%)	TD length (µm)	Difference (%)	PD length (µm)	Difference (%)	NC_PD_	Difference (%)
Col-0	355±39	–	142±20	–	213±19	–	37.3±2.7	–
*mpk6wb*/*lr*	438±47*	23	206±18*	45	232±29**	9	45.7±4.6*	22

Combined data were used from two independent experiments (*n*=24).

*Student’s *t*-test significant differences at *P*<0.001.

**Student’s *t*-test significant differences at *P≤*0.019.

## Discussion

### MPK6 is essential for embryogenesis and root development

The central role that MAPK signalling has over different aspects of plant development is well accepted (Andreasson and Ellis, 2009; Suarez-Rodriguez *et al.*, 2010). However, the dissection of the particular function of each of the MAPK proteins has been difficult due to the partial redundancy among them. Using a genetic strategy, MPK6 has been associated with pathogen resistance (Menke *et al.*, 2004) and anther, inflorescence, embryo, and root development (Bush and Krysan, 2007; Müller *et al.*, 2010; Wang *et al.*, 2010). In particular, with respect to embryo and root development, previous analysis focused on embryo protruding seeds (Bush and Krysan, 2007), short-root seedlings (Müller *et al.*, 2010), and LR development in response to NO treatment (Wang *et al.*, 2010). In this work, we performed a detailed analysis of seed morphology and its correlation with root development in *mpk6* mutants. Three phenotypic classes of seed were identified in the progeny of homozygous *mpk6* plants, including seeds with a normal appearance but bigger than wild-type seeds, seeds with rough coats, and seeds with protruding embryos, each giving rise to seedlings with totally different root growth and development patterns (Figs 1–4). A previous work reported that the *mpk6* mutant displayed a reduced fertility phenotype with variable penetrance depending on growth conditions, but the environmental variable affecting that phenotype remained to be determined (Bush and Krysan, 2007). However, the seed and root phenotypes reported here were reproducible in at least four generations of the progenies of homozygous *MPK6* from two independent null alleles (SALK_073907 and SALK_127507) grown under conditions of 21 °C, long days (16/8h light/dark), 105 µmol m^−2^ s^−1^ of light intensity, and 45–60% of relative humidity at ~1580 m above sea level.

Between 500 and 1000 *Arabidopsis* loci have been related to embryo-defective phenotypes (Meinke *et al.*, 2009), and several of these include members of the MAPK cascade. For example, mutations in the *MPKKK4* (*YDA*) protein kinase gene cause defects in embryo development (http://www.seedgenes.org). Since the identification of *YDA* as a gene required for embryonic cell fates, it has been suggested that a MAPK signalling pathway is involved in *Arabidopsis* embryogenesis (Lukowitz *et al.*, 2004). Interestingly, the *yda/emb71* ethyl methanesulfonate heterozygous mutant, affected in the *YDA* gene (At1g63700), displays a similar embryo-protruding phenotype to that observed in *mpk6* (Lukowitz *et al.*, 2004; Meinke *et al.*, 2009). *MPKKK4* and *MPK6* are components of a common MAPK cascade involved in regulation of the embryo (Bush and Krysan, 2007) and in stomata developmental programmes (Wang *et al.*, 2007). The *yda* mutant has defects in hypophysis development similar to that observed in *mpk6bs* mutants (Fig. 5) (Lukowitz *et al.*, 2004; Meinke *et al.*, 2009). The phenotypes observed during early embryogenesis suggest that MPK6 acts as a repressor of cell proliferation involved in the establishment of embryonic polarity (Fig. 5C). This MPK6 role seems to be maintained throughout development because the *mpk6* mature embryos that achieved complete organogenesis were larger than their wild-type counterparts (Fig. 5I, J). The molecular and cellular mechanisms regulating seed development and size are complex (Sun *et al.*, 2010). Potential targets (transcription factors) and activators (leucine-rich repeat receptor kinases), but not MAPKs were previously involved in that process (Sun *et al.*, 2010). The data from this study further confirm that both YDA and MPK6 are components of a MAPK cascade involved in the regulation of the embryo developmental programme, as already proposed (Bush and Krysan, 2007). The components acting up- and downstream of these MAPKs remain to be identified. In addition, our data support the suggestion that the failure to form a PR and the short-root phenotypes are consequences of *mpk6* mutant embryo development defects. Therefore, without considering the pleiotropic effects caused by embryo defects, the PR phenotype that can be directly associated with the loss of *MPK6* function is a long PR. Notably, this *mpk6* long PR was observed previously (Takahashi *et al.*, 2007), and in the *Arabidopsis* Hormone Database (http://ahd.cbi.pku.edu.cn) *MPK6* is included as one of the 79 genes related to a long-root phenotype.

Previous analysis made on *mpk6* short-root seedlings showed that the loss of *MPK6* function resulted in a slight but significant reduction in the number of LRs, suggesting that MPK6 is a positive regulator of LR formation (Müller *et al.*, 2010). In contrast, the data shown here using only *mpk6* mutants germinated from seeds without embryo damage demonstrated that the longer PRs had increased numbers of LRs and RHs (Figs 4 and 7). These apparent contradictory results could be explained from the different seed classes produced in the *mpk6* mutant progenies. These observations highlight how critical is to perform detailed analyses of the phenotypes associated with a gene mutation in different organs and under strictly controlled growth conditions.

### MPK6 is a negative regulator of primary root growth

The comparisons of the RAM TDs and PDs and the fully elongated cell lengths between wild-type and *mpk6* mutant roots revealed that both cell division and elongation are altered in PRs of *mpk6* mutant (Table 1 and Fig. 8). Moreover, the number of cells in the *mpk6wb/lr* PD was also higher compared with that in the wild type (Fig. 6) and correlated with lower cell-cycle duration in this mutant (Fig. 8), supporting an important role of MPK6 in controlling cell proliferation and suggesting that its loss of function has a direct consequence in the long-root phenotype. For more than a decade, experimental evidence has supported the involvement of MAPKs in the regulation of cell-cycle progression in yeast (Gustin *et al.*, 1998; Strickfaden *et al.*, 2007), animals (Aliaga *et al.*, 1999; Rodríguez *et al.*, 2010), and plants (Calderini *et al.*, 1998; Jonak *et al.*, 2002; Suarez-Rodriguez *et al.*, 2010).

Regulation of plant cell division and growth is associated with microtubule reorganization, which is assisted with the action of microtubule-associated proteins. Previous reports have shown that some microtubule-associated proteins are regulated by reversible phosphorylation through MAPK cascades (Komis *et al*, 2011). For example, a MAPK from *M. sativa* (MKK3) is indispensable for spindle microtubule reorganization during mitosis (Bögre *et al.*, 1999) and the *Arabidopsis* MPK4 has been shown to be essential for the correct organization of microtubules through the phosphorylation of microtubule-associated protein 65-1 (Beck *et al.*, 2010). Additionally, the *Arabidopsis* MPK18 has been demonstrated to interact physically with a dual-specificity MAPK phosphatase (PROPYZAMIDE HYPERSENSITIVE 1/PHS1) to conform to a reversible phosphorylation/dephosphorylation switch that regulates cortical microtubule formation (Walia *et al.*, 2009). Phosphorylation of a MAP3K (NPK1) by cyclin-dependent kinases has been proposed to be critical for the appropriate cytokinesis progression in *Arabidopsis* (Sasabea *et al.*, 2011). Expression of *MPK6* has been shown to be strong in both the RAM PD and TD, specifically during the pre-prophase band and in the phragmoplast, where it controls cell division plane specification (Müller *et al.*, 2010). Epigenetic modifications like methylation or deacetylation of histones have also been suggested to regulate root development (Fukaki *et al.*, 2006; Krichevsky *et al.*, 2009). Interestingly, in animal systems, MAPK mediates histone phosphorylation, which in turns drives acetylation of histone H3, impacting on gene transcription (Clayton *et al.*, 2000). It remains to be addressed whether a similar regulatory mechanism operates in plant systems.

### MPK6 regulates LR initiation

RSA is determined primarily by the spatio-temporal regulation of lateral root initiation events (Bielach *et al.*, 2012). Mutants having increased number of LRs are relatively infrequent compared with those with reduced number of LRs (De Smet *et al.*, 2006), although an increased number of LRs does not necessary indicate an increase in LR initiation (Dubrovsky and Forde, 2012). The participation of MAPK cascades in LR formation was documented by the phenotypes observed in mutants of MPK4 and its upstream activator MEKK1-1, both displaying from a slight to a severe reduction in LR density (Nakagami *et al.*, 2006; Su *et al.*, 2007). Previous studies have also shown that MPK3 and MPK6 are activated in response to the same signals as MEKK1/MPK4, supporting a possible role of these kinases in the LR development programme (Ichimura *et al.*, 2006; Suarez-Rodriguez *et al.*, 2007). However, our results demonstrated that the role of MPK6 in LR development is opposite to that of MPK4 (and MEKK1). The observation that the *mpk6wb/lr* long PR phenotype is accompanied by an increase in LR initiation (Figs 4C, D and 6), fully demonstrated that MPK6 acts as a negative regulator of LR initiation. The clearest examples of increased LR initiation are the mutants related to auxin homeostasis and signalling (Zhao, 2010). CEGENDUO, a subunit of SCF E3 ligase, has a negative role in auxin-mediated LR formation (Dong *et al.*, 2006). MAPK cascades have been found to directly or indirectly affect auxin signalling (Mockaitis and Howell, 2000; Takahashi *et al.*, 2007), which could alter LR formation. Cytokinin is a negative regulator of LR initiation. Decreased endogenous cytokinin concentration in protoxylem-adjacent pericycle cells results in increased LR initiation; in contrast, when the cytokinin concentration in these cells is increased, LR initiation is repressed (Laplaze *et al.*, 2007). In this context, the *mpk6* mutant shows a phenotype of decreased endogenous cytokinin content. Altogether, these findings highlight the complexity of the MAPK cascades in root morphogenesis. However, an increase in cell production in the PR meristem and increased LR initiation in *mpk6wb/lr* both indicate a link between cell proliferation and its regulation by MPK6. Stress and development responses are tightly coordinated by MPKs, but their interaction is still poorly understood. As few research studies have focused on the interplay between development and environmental stresses, our findings highlight the power of studying root processes in terms of unravelling MPK signalling interactions.

### MPK6 is important for RH formation

Our data also demonstrated that MPK6 is a negative regulator of RH development, as its mutation resulted in an increase in the number and size of RHs (Fig. 7). Multiple cellular factors regulate RH growth and development, including vesicle exocytosis, calcium (Ca^2+^) homeostasis, reactive oxygen species and cytoskeleton modifications (Cardenas, 2009). Ca^2+^ is a universal second messenger, which, through interactions with Ca^2+^ sensor proteins, performs important roles in plant cell signalling (Batistič and Kudla, 2012). These sensor proteins include calmodulins, calmodulin-like proteins, Ca^2+^-dependent protein kinases, calcineurin B-like proteins, and their interacting kinases, among others. Several genes implicated in RH differentiation have been identified; one of them, *OXIDATIVE SIGNAL INDUCIBLE 1* (*OXI1*) from *Arabidopsis*, is required for MPK6 activation by reactive oxygen species (Rentel *et al.*, 2004). The MPKKK1 (MEKK1) also has been involved in reactive oxygen species homeostasis (Nakagami *et al.*, 2006) and apparently, together with MKK2 and MPK4/MPK6, constitutes a MAPK cascade that participates in several stress responses (Ichimura *et al.*, 2000). In alfalfa, stress-induced MAPK (SIMK), an *Arabidopsis MPK6* orthologue, performs an important role in RH tip growth (Šamaj *et al.*, 2002). The alfalfa SIMK protein seems to be a positive regulator of RH growth, as treatment of plants with the MAPK inhibitor UO126 resulted in aberrant RHs, whereas the overexpression of SIMK in tobacco induced a rapid growth of these cells (Šamaj *et al.*, 2002). These results contrast with our observations of the function of MPK6 and highlight a specific role of each member of the MAPK cascade in a particular developmental process.

### Possible role of MPK6 in hormone responses

As the precise mechanism underlying the root developmental alterations in *mpk6* seedlings is still unknown, we hypothesized that *mpk6wb/lr* root architectural phenotypes might result from altered responses to auxins or cytokinins, as these hormones control RSA (Perilli *et al.*, 2012). Thus, to determine whether MPK6 could affect PR responses to auxins or cytokinin, we evaluated the PR growth of wild-type and *mpk6wb/lr* mutant seedlings in response to the exogenous addition of IAA and kinetin. We observed that, at low concentrations of IAA (0.03–0.125 µM) and kinetin (0.25–2 µM), *mpk6wb/lr* was slightly insensitive and slightly hypersensitive, respectively, to the inhibitory effects of these hormones on PR growth. However, these differences were not clear at higher concentrations (0.25–0.5 µM IAA and 4–16 µM kinetin) of both hormones (Supplementary Fig. S4 at *JXB* online). These assays showed that *mpk6wb/lr* PR is not insensitive to the exogenous addition of these two plant growth regulators, suggesting that the observed root length differences in the *mpk6* mutant cannot be explained by different sensitivities to auxin or cytokinins. This observation agrees with the finding that the root growth-inhibition response to several hormones of the MPKKK mutant *yda*, which acts upstream of MPK6, is normal (Lukowitz *et al.*, 2004).

In summary, the results presented here indicate that MPK6 is a negative regulator of at least three developmental programmes in the root, namely PR growth, LR formation, and RH development, which probably occurs through regulation of cell division and elongation processes. Understanding the signalling events regulated by MPK6 activity during root development will ultimately require identification of the up- and downstream components, as well as the signal (or combination of signals) turning on and off phosphorylation of the MAPK cascade and impacting on RAM behaviour.

## Supplementary data

Supplementary data are available at *JXB* online.

Supplementary Methods.

Supplementary Figure S1. *mpk6* is a null mutant.

Supplementary Figure S2. *mpk6* seed phenotypes are stable.

Supplementary Figure S3. *mpk6* siliques are shorter than wild type and contain many aborted seeds.

Supplementary Figure S4. Effect of auxin and cytokinins on primary root growth.

Supplementary Data

## Abbreviations:

CPR: cell production rate
DAG: days after germination
IAA: indole-3-acetic acid
LR: lateral root
LRP: lateral root primordium
MAPK: mitogen-activated protein kinase
MPK6: *Arabidopsis thaliana* mitogen-activated protein kinase 6
NO: nitric oxide
PD: proliferation domain
PR: primary root
RAM: root apical meristem
RH: root hair
TD: transition domain.
