# Supplementary material for: Arabidopsis thaliana mitogen-activated protein kinase 6 is involved in seed formation and modulation of primary and lateral root development
Source: J Exp Bot. 2013 Nov 11;65(1):169–83. doi: 10.1093/jxb/ert368 (PMC3883294; doi:10.1093/jxb/ert368)
Supplement: Supplementary Data [file supp_65_1_169__index.html]

Arabidopsis thaliana mitogen-activated protein kinase 6 is involved in seed formation and modulation of primary and lateral root development — Arabidopsis thaliana mitogen-activated protein kinase 6 is involved in seed formation and modulation of primary and lateral root development — Supplementary Data 

# *Arabidopsis thaliana* mitogen-activated protein kinase 6 is involved in seed formation and modulation of primary and lateral root development

## Supplementary Data

Data files

**Files in this Data Supplement:**

- Supplementary Data - Supplementary Data
