# Supplementary material for: Arabidopsis thaliana mitogen-activated protein kinase 6 is involved in seed formation and modulation of primary and lateral root development
Source: J Exp Bot. 2013 Nov 11;65(1):169–83. doi: 10.1093/jxb/ert368 (PMC3883294; doi:10.1093/jxb/ert368)
Supplement: Supplementary Data [file supp_ert368_jexbot104372_file001.pdf]

Arabidopsis thaliana **MAP kinase 6 mutation causes three different seed phenotypes correlated with alterations in cellular processes affecting root architecture**

López-Bucio J. S., Dubrovsky, J. G., Raya-González J., Ugartechea-Chirino, Y., LópezBucio J., de Luna-Valdés, L. A., Ramos-Vega, M., León, P. and Guevara-García, A. A[1].

Supplementary Methods

Plant genotyping

Total RNA was isolated using TRIZOL® (Invitrogen™, Carisbad, CA) from freezing tissue and the first-strand cDNA was synthesized using 3 µg of RNA in a volume of 30 µL containing 50 mM Tris-HCl, pH 8.3, 75 mM KCl, 3 mM $MgCl_2$, 5 mM DTT, 2 mM deoxynucleotide triphosphate mixture, 5 mM oligo(dT) primer, 10 units of RNase inhibitor (Invitrogen™), and 100 units of Moloney murine leukemia virus reverse transcriptase (Invitrogen™) for 1 h at 42 ºC. To inactivate the enzyme the samples were incubated at 92 ºC by 10 min. One microliter of the first-strand reaction was used for the genotypic analysis of the plant lines under study. The amplification reaction was performed containing 0.2 mM of each deoxynucleotide triphosphate, 0.5 mM of each primer, 10 mM Tris-HCl, pH 8.8 at 25 ºC, 50 mM KCl, 0.08% Nonidet P-40, 2.5 mM $MgCl_2$, and 1 unit of Taq DNA polymerase (MBI/Fermentas, Hannover, MD). Thirty cycles of amplification were performed in a PTC-200 Peltier thermal cycler (MJ Research, Waltham, MA), each having 30 s of denaturation at 94 ºC, 40 s of annealing at 56 ºC, and 1 min of extension at 72 ºC. The specific set of primers for each amplification reaction was as follows: 5'-CTTCCGCCGTAAAAGC-3' (forward) and 5'-GTCCCAGCACCACAGG-3' (reverse) for *UBI6* (At2g47110), 5'-ATCTTTATGGAGCTTATG-3' (forward) 5'-CCGTATGTTGGATTGAG-3' (reverse) for *MPK3* (At3g45640) and 5'-CGAGTCACTTCTGAGAG-3' (forward) and 5'-TTGCTGATATTCTGG-3' (reverse) for *MPK6* (At2g43790). PCR products were analyzed on 1% agarose gels.

Protein extraction and kinase assays

Seedlings were ground in liquid nitrogen, homogenized in buffer containing 250 mM sorbitol, 50 mM HEPES-BTP (pH 7.8), 10 mM NaF, 5 mM DTT, 1 mM EDTA, 1 mM KCl, 1 mM Na_3VO_4, 1 mM PMSF and 40 µg/ml of protease inhibitor cocktail (Roche Applied Science, Indianapolis, IN), then centrifuged at 12,000 x g 20 min at 4ºC. The crude extracts were stored at –70ºC. Protein concentration in the extracts was estimated with Bradford protein assay kit (Bio-Rad, Hercules, CA) using BSA as a standard. The in-gel kinase assay was performed as previously described (Zhang & Klessig 1997). Briefly, 50µg of protein extracted from plant tissue were fractionated on a 10% SDS-polyacrylamide gel containing 0.25 mg/ml myelin basic protein (MBP, Sigma) as substrate for the kinases. After electrophoresis, the gel was washed three times with 25 mM Tris (pH 7.5); 0.5 mM DTT; 0.1 mMNa3VO4; 5mM NaF; 0.5 mg/ml BSA; 0.1% (vol/vol) Triton X-100 for 30 min each at room temperature. Proteins in the gel were then renatured by incubating the gel in 25 mM Tris (pH 7.5); 1 mM DTT; 0.1 mM Na_3VO_4 and 5 mM NaF at 4°C overnight, with three changes of buffer. The kinase reactions were then carried out by incubating the gel in 30 ml of buffer containing 25 mM Tris (pH 7.5); 2 mM EGTA; 12 mM $MgCl_2$; 1mM DTT; 0.1 mM Na_3VO_4; 200 nM ATP, and 50µCi of [γ-^{32}P]ATP (>4,000 Ci_mmol; 1 Ci = 37 GBq), for 60 min at room temperature. To

[1] aguevara@ibt.unam.mx

remove free ^{32}P, the gel was extensively washed at room temperature with several changes of 5% (wt/vol) trichloroacetic acid and 1% (wt/vol) NaPPi until ^{32}P-radioactivity in the wash solution was barely detectable. The gel was dried under vacuum on Whatman 3MM paper and used to expose a Kodak XAR-5 film. Prestained size markers (Bio-Rad, Hercules CA) were used to calculate the size of kinases. As loading control 20μg of proteins from the same extracts used for kinase assays were fractionated in a 10% SDS-polyacrylamide gel and stained with Coomassie Brilliant Blue dye.

***β*-glucuronidase histochemical activity**

Expression of the *GUS* reporter gene (Jefferson *et al.,* 1987) was detected by incubating *Arabidopsis* imbibed seeds or *in vitro* grown seedlings, in histochemical assay buffer (100 mM NaH_2PO_4 and Na_2HPO_4 mix, pH 7; 0.5 mM potassium ferrocyanide; 0.5 mM potassium ferricyanide; Na_2EDTA pH 8.0; 0.1% Triton X-100 and 0.1% 5-bromo-4-chlorium-3-indolyl-b-D-glucuronic acid), at 37 ºC during the indicated time. Chlorophyll was removed with 70% ethanol for several hours. Seedlings were cleared according to Malamy and Benfey (1997) with some modifications (Dubrovsky *et al.,* 2006), and then mounted in 50% glycerol on microscope slides. Histochemically stained or unstained seeds and seedlings were analyzed using stereoscopic (Nikon SMZ1500) and transmission (Nikon EclipseE600) microscopes equipped with a digital camera (Nikon SIGHT DS-Fi1c, Nikon Corporation, Tokyo, Japan).

Figure Legends

Supplemental Figure S1. *mpk6* is a null mutant.

A) Genotyping of the plants under study. RT-PCR analysis of RNAs from wild-type (Wt, Col-0), *mpk3-1* (SALK_151594), *mpk6-2* (SALK_073907) and *mpk6-3* (SALK_127507) lines, previously characterized (Liu and Zhang, 2004; Wang *et al.,* 2007). The gene fragment amplified in each case is indicated. UBI6 corresponds to the ubiquitin-6 (AT2G47110) *Arabidopsis* gene. All the PCRs were made with the same cDNA from the indicated plant lines. B) In-gel kinase assays were conducted with total protein extracts from wild-type (Wt/Col-0), F1 progeny of *mpk6* x Col-0, F2 seedlings from three seed phenotypes (*mpk6bs*, *mpk6rs*, *mpk6wb*) and *mpk3*, 6DAG seedlings. Notice that whereas MPK6 activity is evident on heterozygous F1 progeny, no MPK6 activity could be detected on *mpk6* seedling with seed phenotypes described here. A Coomassie Brilliant Blue Gel (CBBG) is shown as loading control.

Supplemental Figure S2. *mpk6* seed phenotypes are stable.

A) Representative *mpk6* x *pABI4::GUS* silique containing the three *mpk6* classes of seeds (wb/wild-type bigger; rs/raisin-like and bs/burst seed) is shown. B) Segregating seed phenotypes and histochemical GUS activity on the F3 *mpk6* x *pABI4::GUS* seed protruding embryos are shown. In the picture the GUS positive burst-seeds are over represented, but each phenotype was present in a proportion similar to that observed in the homozygous *mpk6* mutant line (~70, ~23 and ~7%, respectively). Scale bars = 1 mm.

Supplemental Figure S3. *mpk6* siliques are shorter than wild-type and contain many aborted seeds. Comparison between wild-type (Wt, Col-0) and *mpk6* homozygous line of: A) The length of siliques, B) The number of seeds per silique and C) Seeds aborted per silique. The Error bars represent SE from 10 siliques along the stem of 5 independent plants (*n*= 50 for each). Asterisk marks Student's t-test significant differences at *P* indicated. D) Representative photograph of wild-type (Wt, Col-0) and *mpk6* siliques. An abortion event, apparently frequent on *mpk6* siliques, is highlighting (arrow). Scale bars = 1 mm.

Supplemental Figure S4. Effect of auxin and cytokinins on primary root growth
Primary root length inhibition caused by the indicated concentrations of: A) Idol-3Acetic Acid (IAA) and B) kinetin over wild-type (Wt, Col-0) and *mpk6wb/lr* seedlings**.** Data were recorded from 8 DAG seedlings growing in media supplemented with the corresponding hormone. The percentages of root growth inhibition were calculated taken PR length of seedlings growing in basal medium as 0% of inhibition. Values are mean ± Standard Error (*n* = 15). Different letters represent Tukey's post-hoc test significant differences (*P*≤0.05). The experiment was repeated three times with similar results**.**

Supplementary Figures

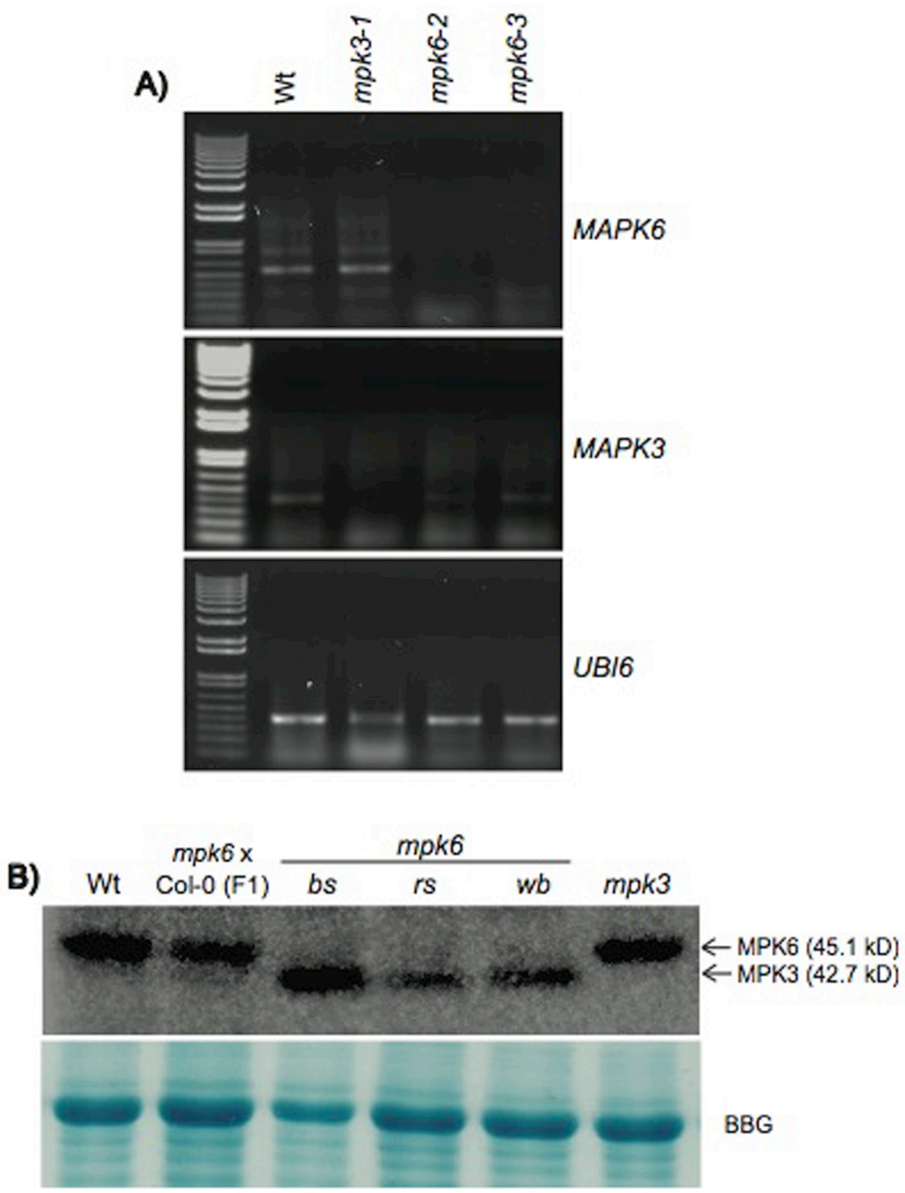

Supplemental Figure S1.

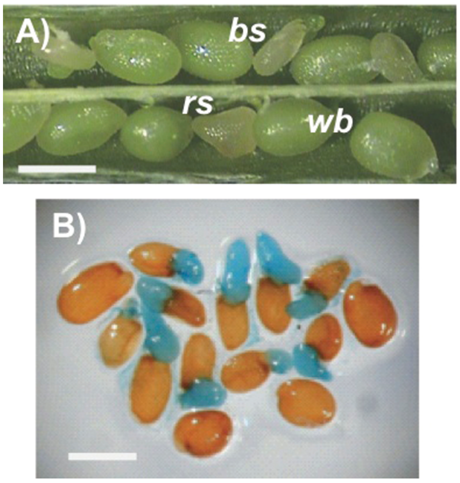

Supplemental Figure S2.

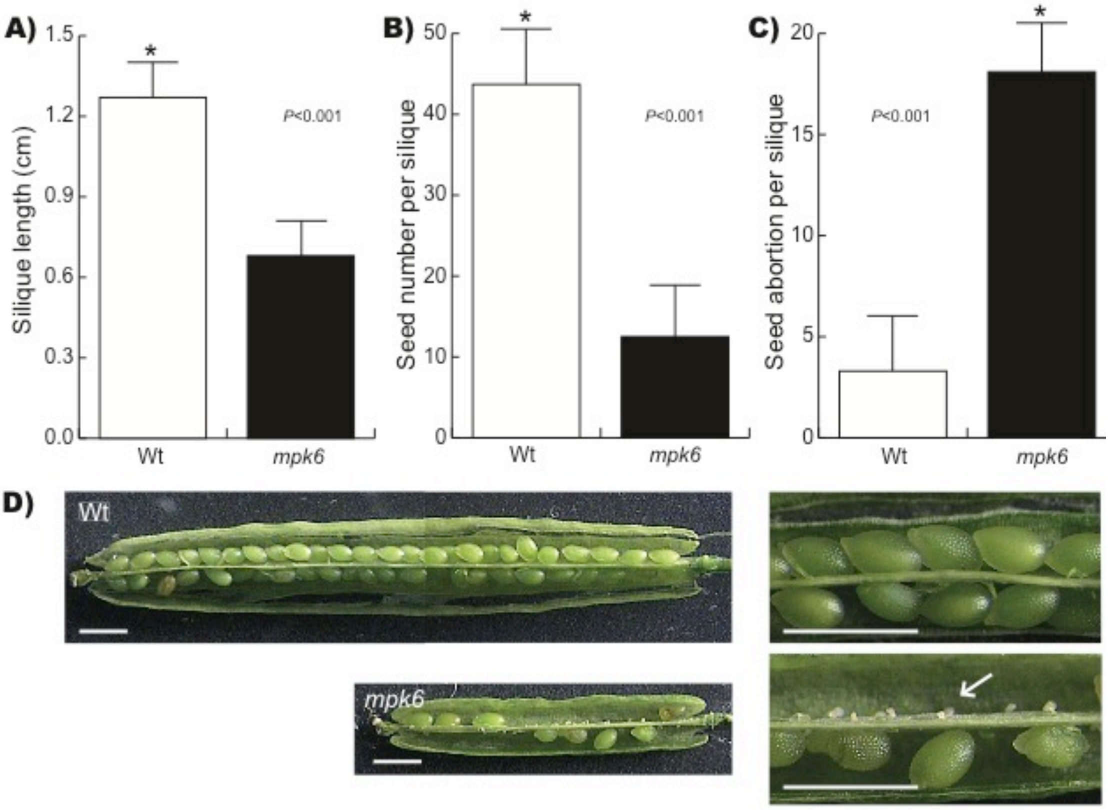

Supplemental Figure S3.

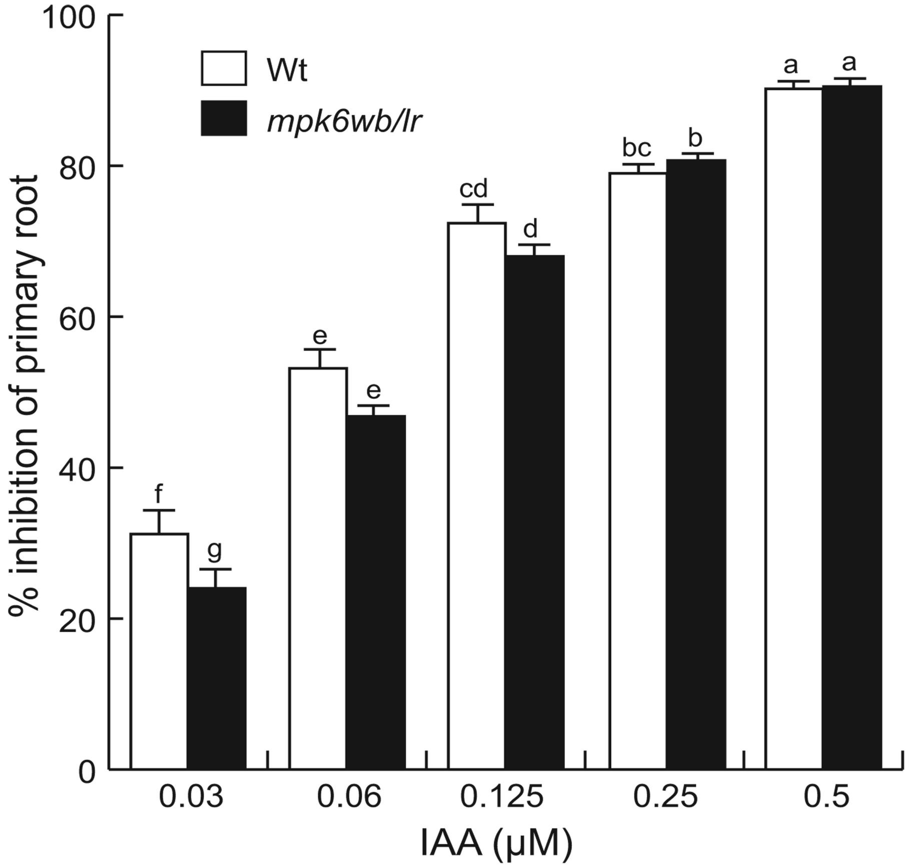

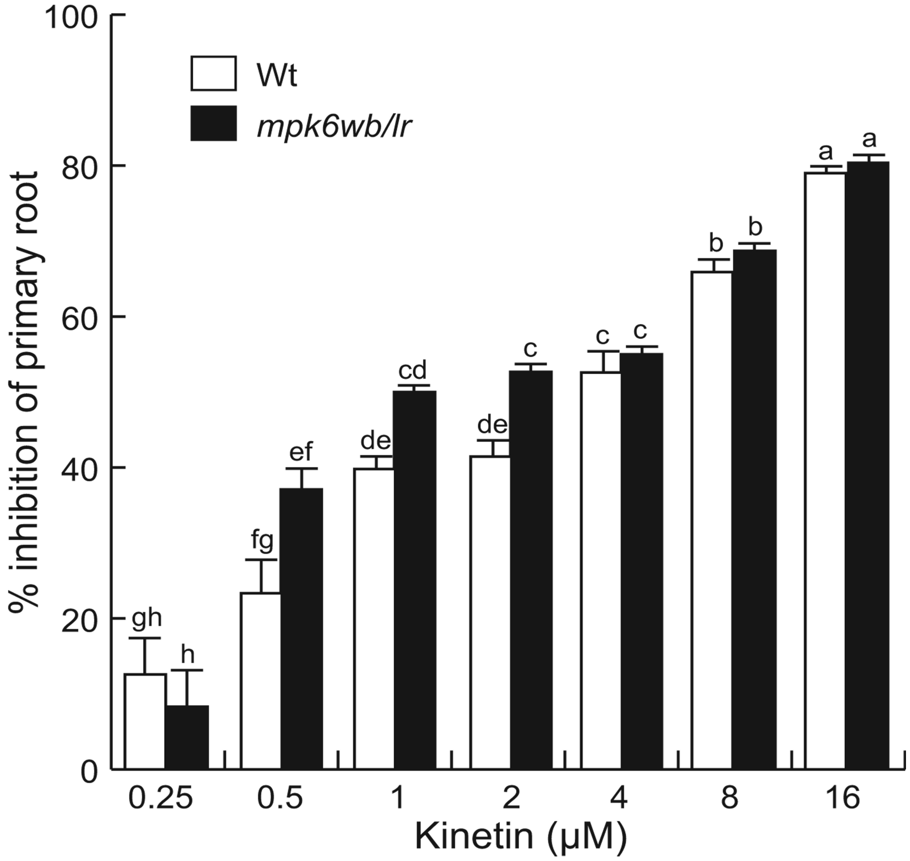

Supplemental Figure S4.
